# Supplementary material for: Nr4a1 modulates inflammation and heart regeneration in zebrafish
Source: Development. 2025 Jul 11;152(20):dev204395. doi: 10.1242/dev.204395 (PMC12276807; doi:10.1242/dev.204395)
Supplement: Supplementary information [file develop-152-204395-s1.pdf]

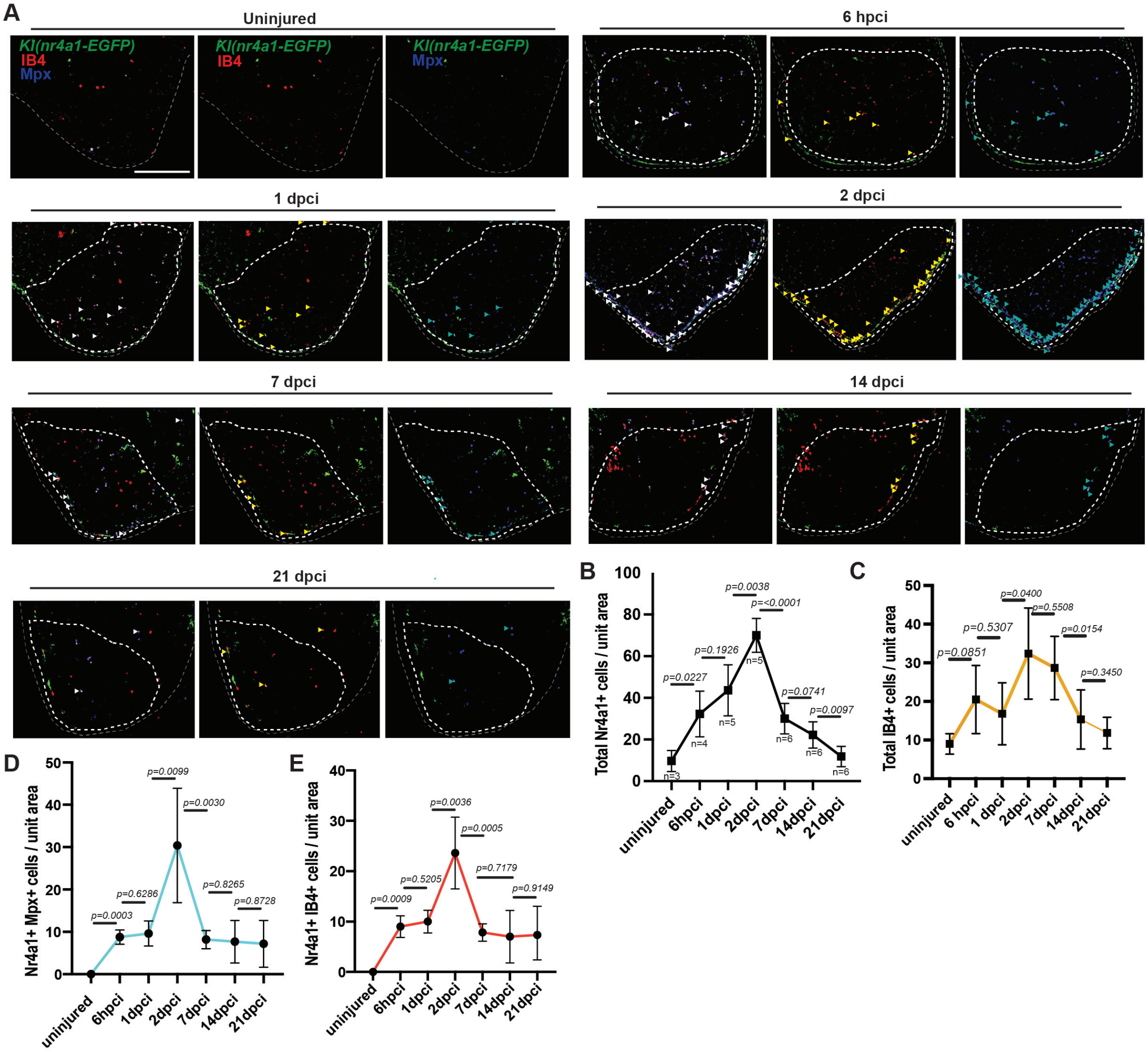

Fig. S1. Dynamics of Nr4a1+ cells in comparison with neutrophils and macrophages after cardiac injury.

(A) Fluorescence signals from immuno-labeled Nr4a1 positive cells (GFP; green), neutrophils (Mpx; blue), and macrophages (IB4; red) in and near the wound area at multiple time points after injury, respectively. White dash lines mark the injured area. (B, C) Temporal dynamics of Nr4a1+ cell or IB4+ macrophage numbers across different stages after injury, respectively. (D, E) Temporal dynamics of double positive cell numbers across different stages after injury, respectively. White arrowheads in A indicate the triple-labeled signals (Left); Yellow arrowheads in A indicate the Nr4a1&IB4 double-double labeled signals (Middle); Cyan arrowheads in A indicate the Nr4a1&Mpx double labeled signals (Right); White asterisk in A (Left) indicate the Mpx&IB4 double positive signals; The entire image in A represents a unit area. A two-tailed unpaired t-test is used. P-values<0.05 were considered statistically significant. Scale bar =275 µm.

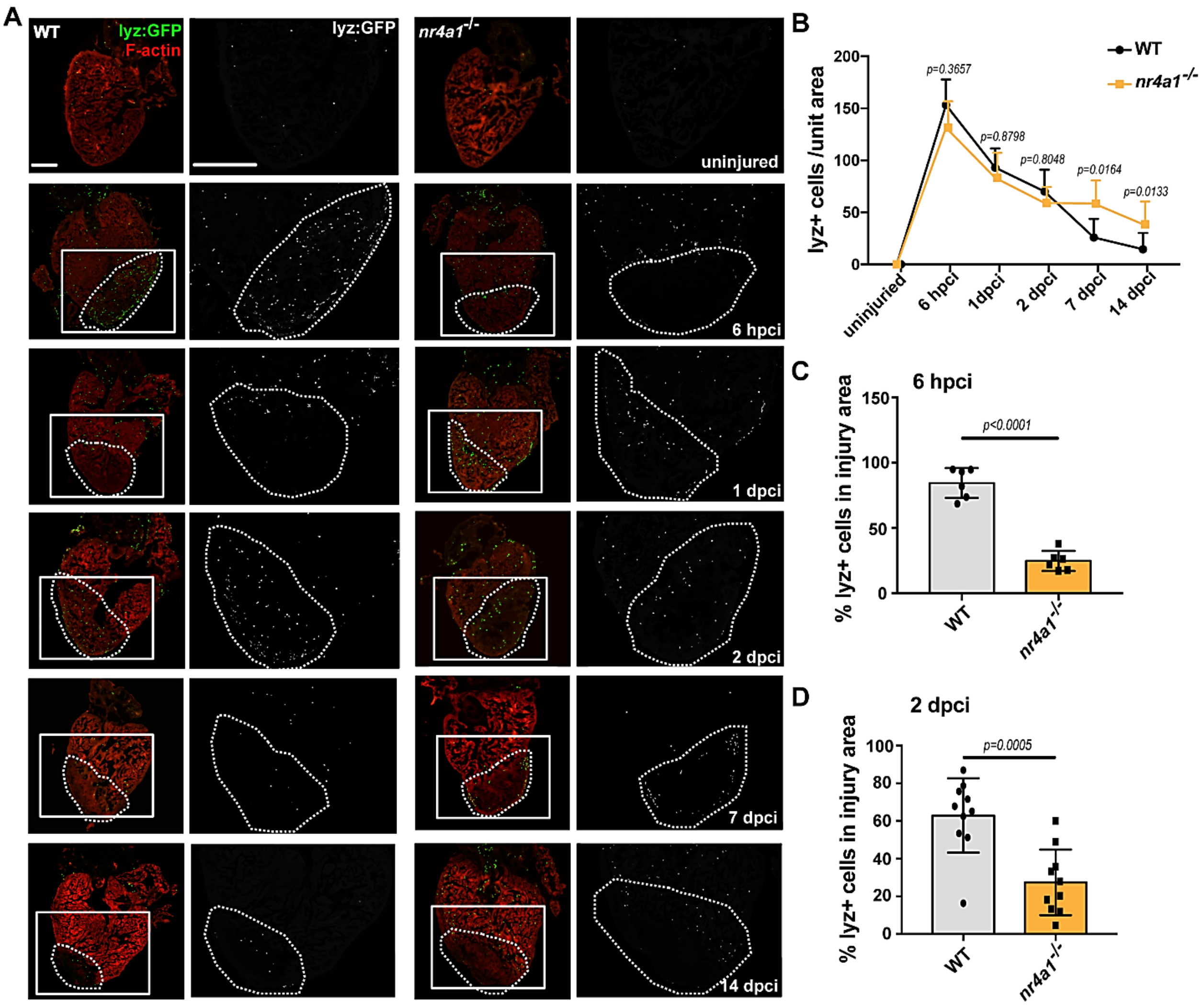

Fig. S2. Distribution of *lyz:eGFP*+ neutrophils in *nr4a1* mutant

(A) Neutrophil distribution in wt and *nr4a1* mutant at different stages after injury. (B) Temporal dynamics of neutrophil number across different stages after injury. The sample number 'n' is marked in the plot. (C, D) Percentage of neutrophil number in the wounded sites per unit area. Symbols show the sample number. Dash lines mark the wounded area. Box regions mark the approximate positions for quantification. A two-way ANOVA with Sidak test for multiple comparison correction is used in panel B. Two-tailed unpaired t-test is used in panels C and D. P-values<0.05 were considered statistically significant. Scale bar: A=275 µm.

Development • Supplementary information

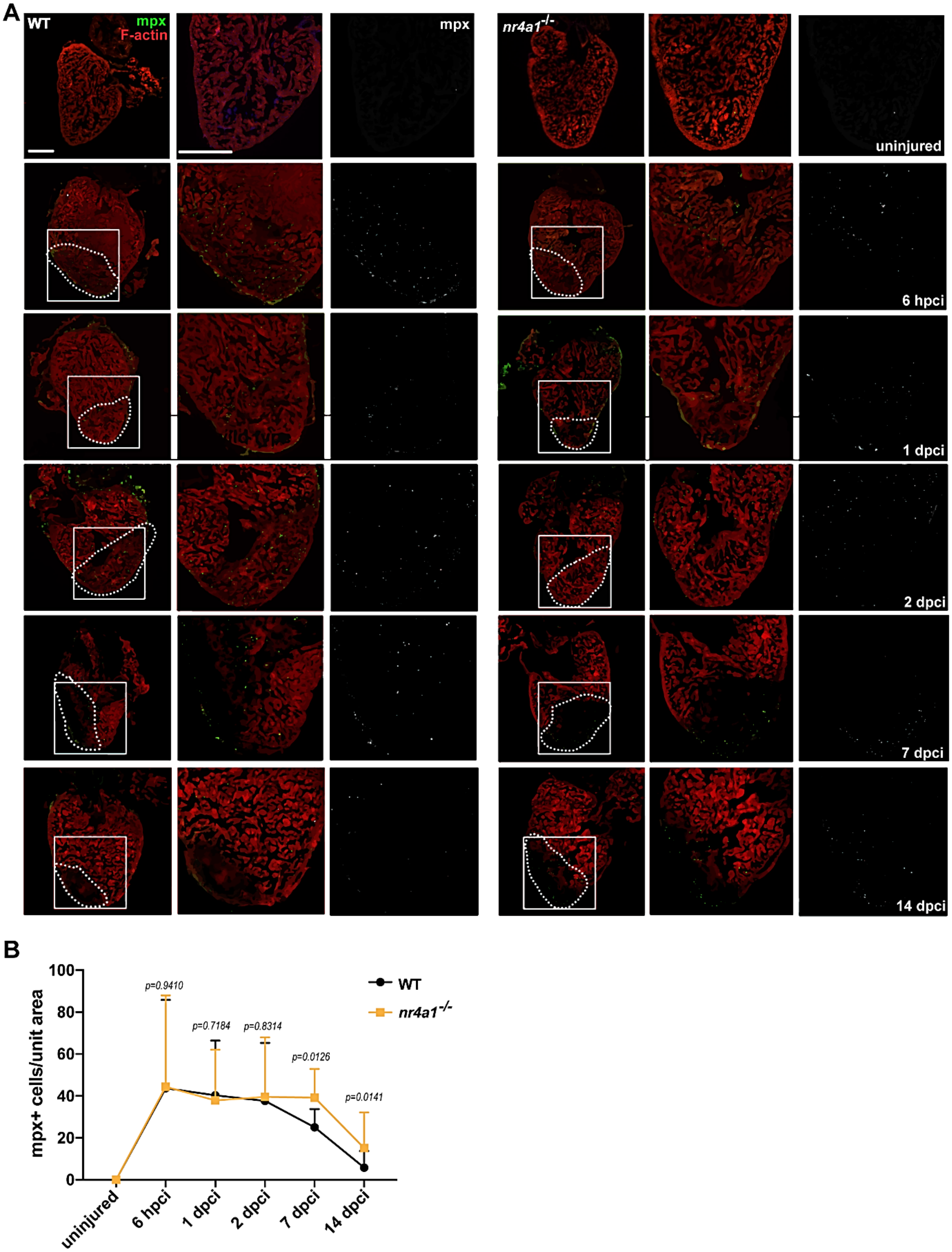

Development • Supplementary information

Fig. S3. Distribution of Mpx+ neutrophils in *nr4a1* mutant

(A) Neutrophil distribution in wt and *nr4a1* mutant at different stages after injury. (B) Temporal dynamics of neutrophil number across different stages after injury. The sample number ‘n’ is marked in the plot. Dash lines mark the wounded area. Box regions mark the approximate positions for quantification. A two-way ANOVA with Sidak test for multiple comparison correction is used in panel B. P-values<0.05 were considered statistically significant. Scale bar: A=275 µm.

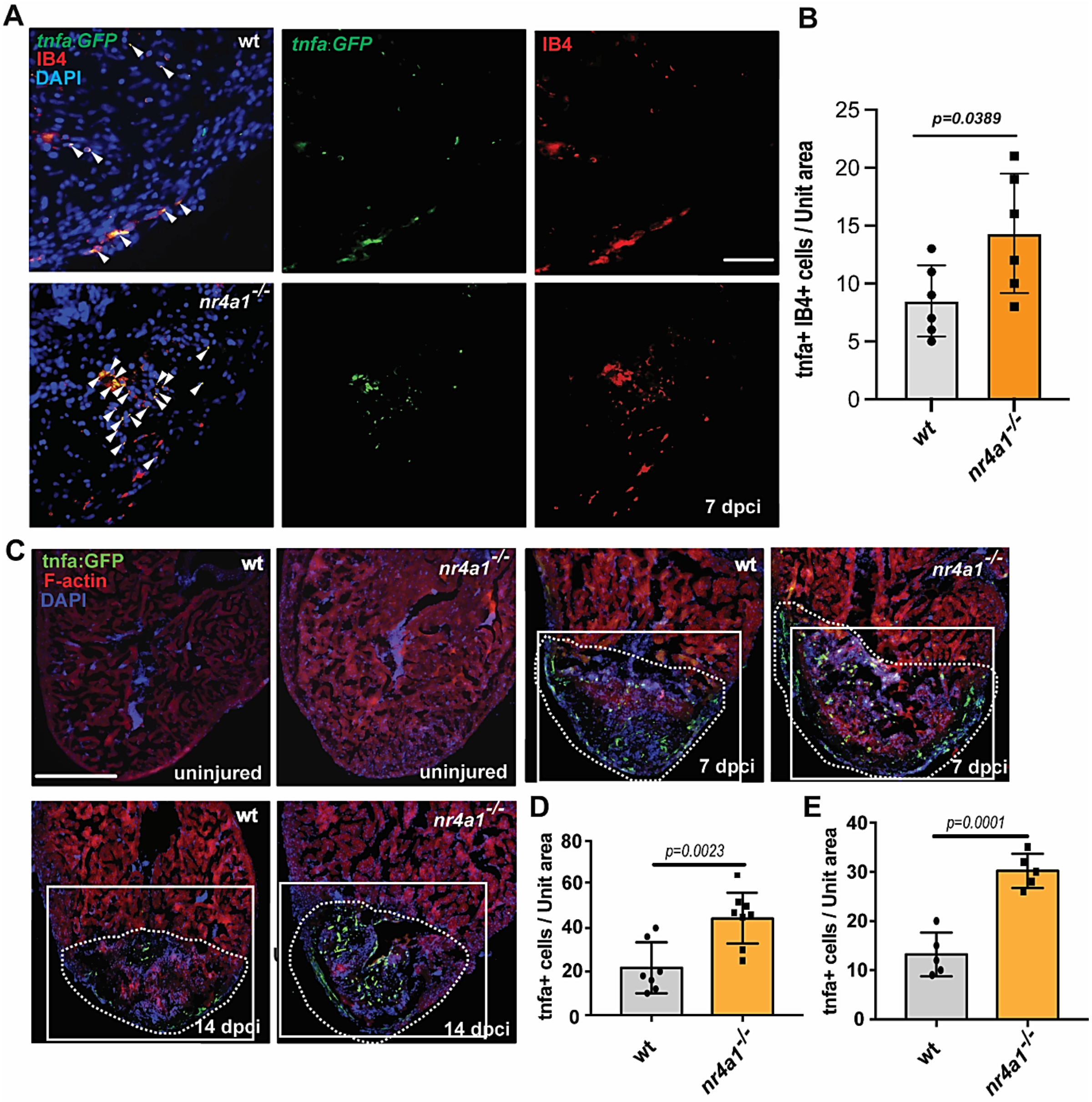

Fig. S4. Increased number of *tnfa:eGFP*+ inflammatory macrophages in *nr4a1* mutant

(A) Immunohistochemistry for GFP coupled with IB4 staining on 7 dpci sections from wt and *nr4a1* mutant fish carrying *tnfa:gfp* transgene. (B) Quantification of the number of tnfa+/IB4+ cells in the injured area. Arrows mark the tnfa+/IB4+ cells. (C) Distribution of inflammatory macrophages in wt and *nr4a1* mutant at different stages. (D) Quantification of the number of inflammatory macrophages per unit area at 7 dpci. (E) Quantification of the number of inflammatory macrophages per unit area at 14 dpci. Dash lines mark the wounded area. Box regions in C mark the approximate positions for quantification. The entire image in A represents a unit area. A two-tailed unpaired t-test is used. P-values<0.05 were considered statistically significant. Scale bar: A=25 µm. C=275 µm.

Development • Supplementary information

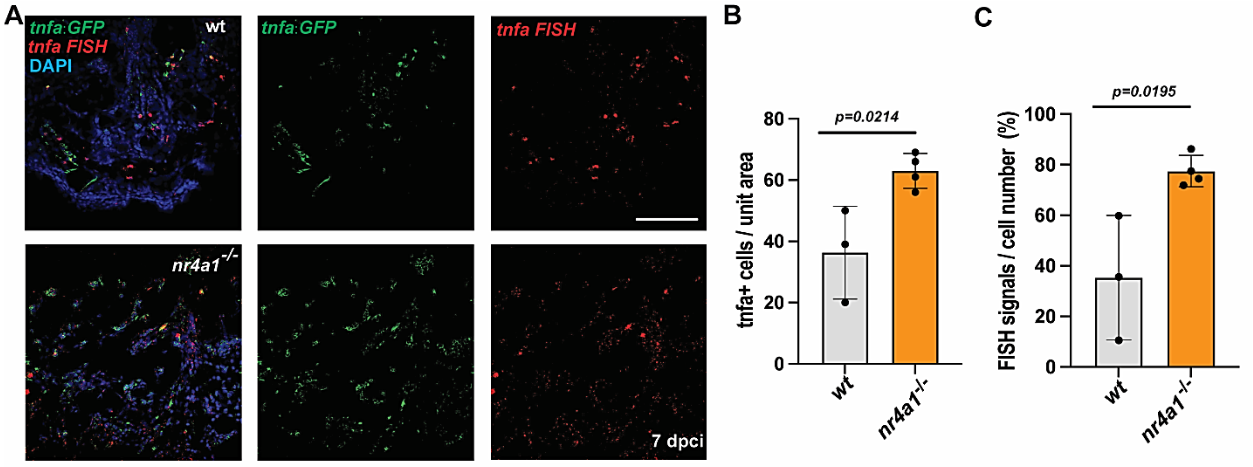

Fig. S5. Increased *tnfa* mRNA signals in *nr4a1* mutant

(A) Concurrent RNAscope *in situ* hybridization for *tnfa* and immunostaining for GFP on 7 dpci sections from wt and *nr4a1* mutant fish carrying *tnfa:eGFP* transgene. (B, C) Quantification of the number of *tnfa:eGFP*+ cells and the relative *tnfa* mRNA signals normalized to *tnfa:eGFP*+ cells, respectively. A two-tailed unpaired t-test is used. P-values<0.05 were considered statistically significant. Scale bar=75 µm.

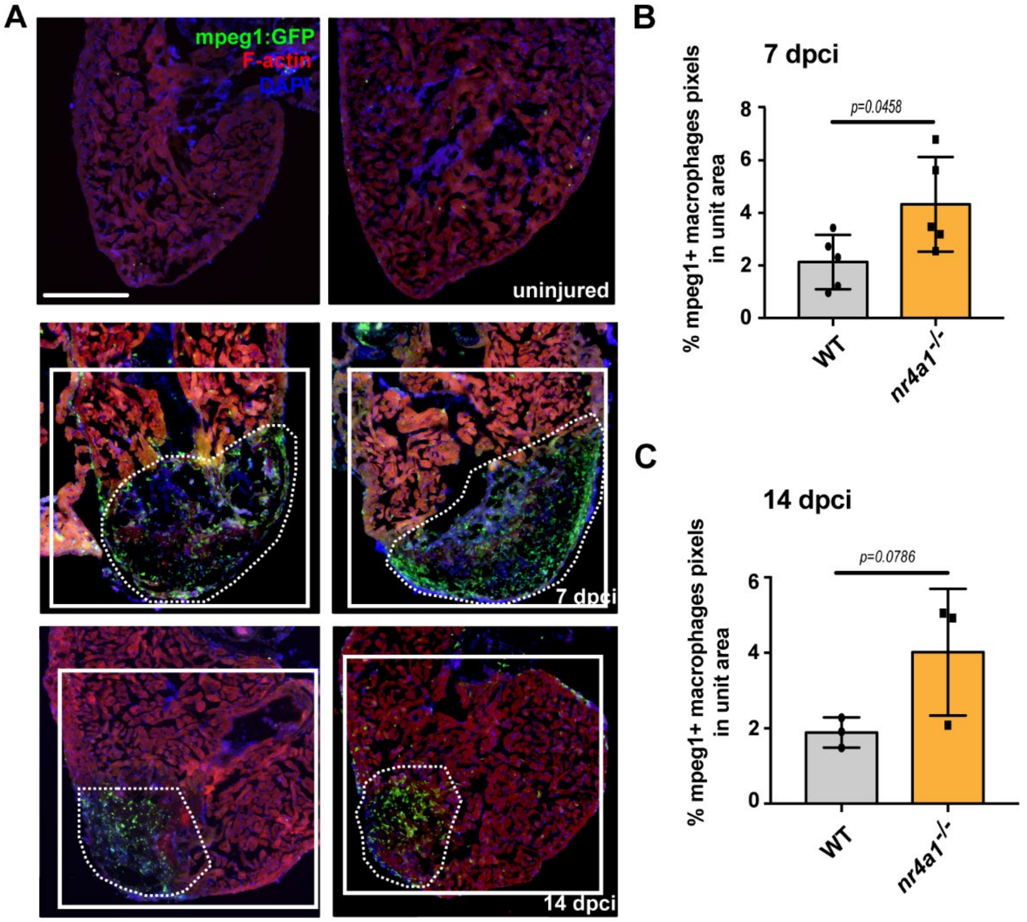

Fig. S6. Increased number of *mpeg1:eGFP*+ macrophages in *nr4a1* mutant

(A) Macrophage distribution in wt and *nr4a1* mutant at different stages from heart sections. (B) Quantification of macrophage number per unit area at 7 dpci. (C) Quantification of macrophage number per unit area at 14 dpci. Dash lines show the wounded area. Box regions show the approximate positions for quantification. A two-tailed unpaired t-test is used. P-values<0.05 were considered statistically significant. Scale bar: A=275 µm.

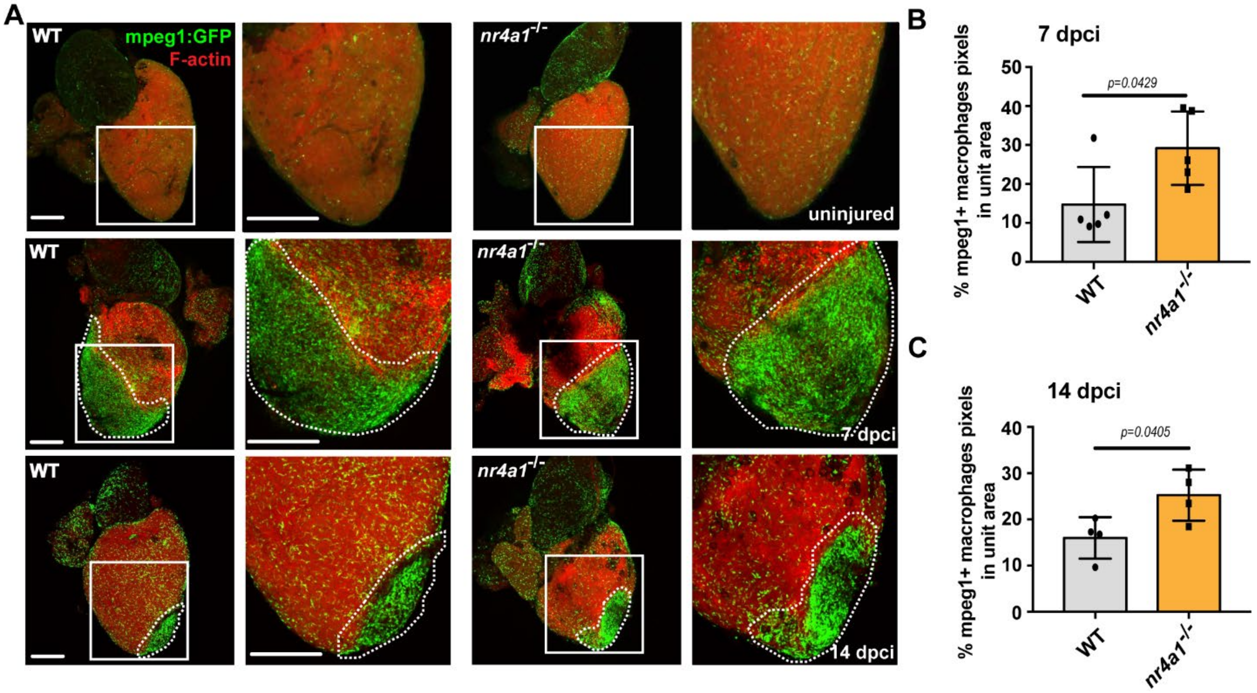

Fig. S7. ***mpeg1: eGFP*+ macrophages in *nr4a1* mutant hearts**

(A) Macrophages distribution in wt and *nr4a1* mutant at different stages from whole heart samples. (B) Quantification of macrophage number per unit area at 7 dpci. (C) Quantification of macrophage number per unit area at 14 dpci. Dash lines mark the wounded area. Box regions mark the approximate positions for quantification. A two-tailed unpaired t-test is used. P-values<0.05 were considered statistically significant. Scale bar: A=275 μm.

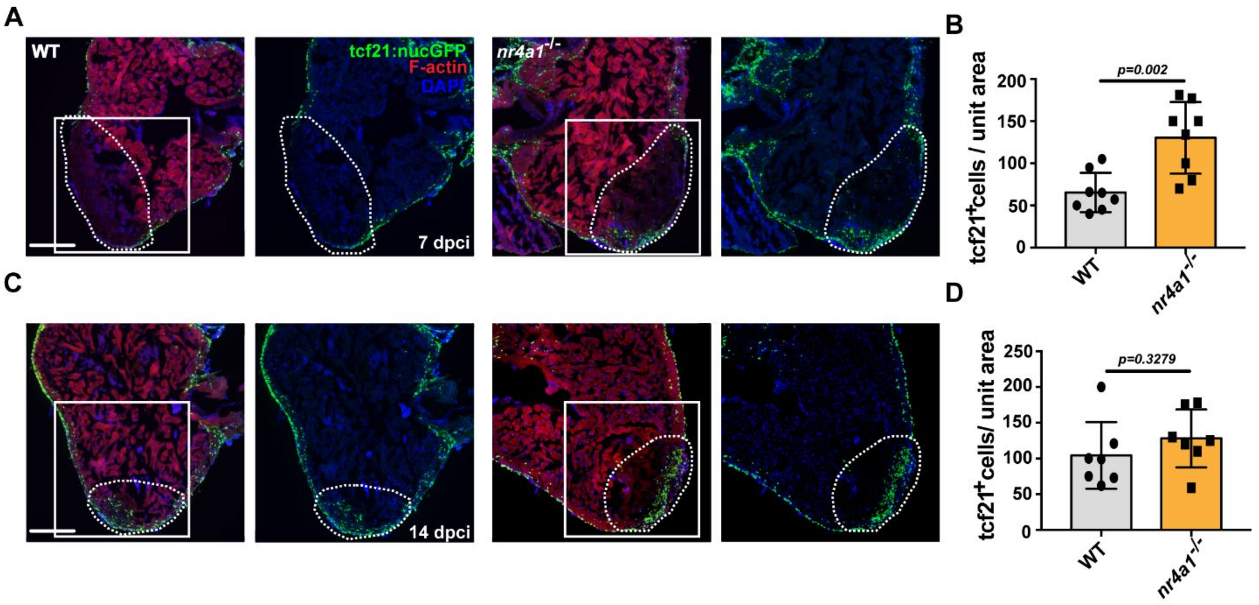

Fig. S8. Distribution of fibroblasts at 7 and 14 dpci after injury

(A, C) Distribution of fibroblasts in the cardiac section of wt and *nr4a1* mutant at 7 and 14 dpci. (B, D) Quantification of fibroblasts per unit area in the wt and *nr4a1* mutant heart at different stages post-injury. Symbols show the individual sample number. A two-tailed unpaired t-test is used. P-values<0.05 were considered statistically significant. Scale bar: A, C=300 µm.

Development • Supplementary information

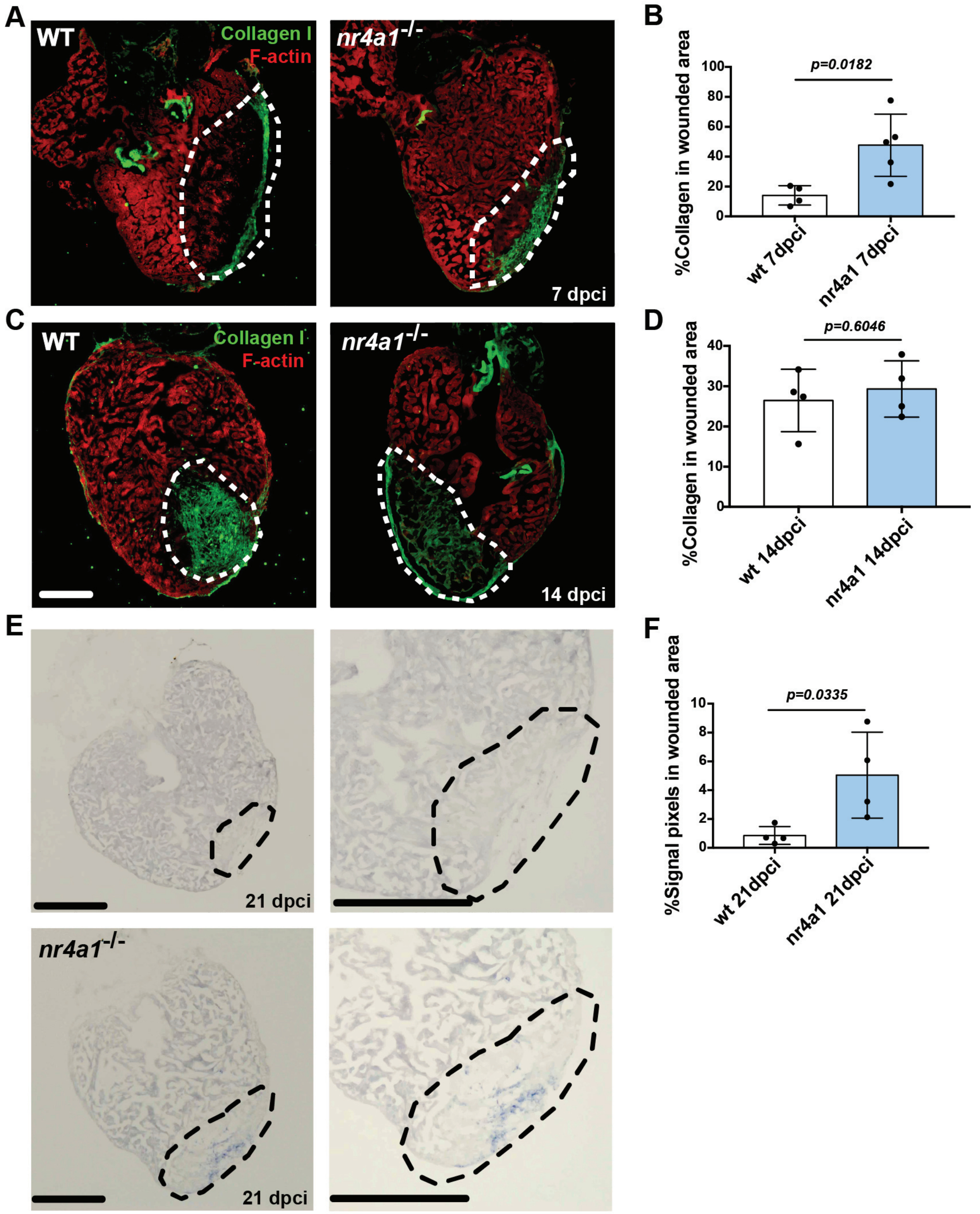

Fig. S9. Collagen deposition and *postnb* expression in *nr4a1* mutant

(A, C) Collagen deposition, as revealed by collagen I antibody staining, in wt and *nr4a1* mutant at 7 dpci and 14 dpci, respectively. (B, D) Quantification of collagen deposition in the wounded area of wt and *nr4a1* mutant hearts at different stages post-injury. (E) *In situ* hybridization of *postnb* in wt and *nr4a1* mutant at 21 dpci. (F) Quantification of *postnb* expression in wt and *nr4a1* mutant at 21 dpci. Symbols show the individual sample number. A two-tailed unpaired t-test is used. P-values<0.05 were considered statistically significant. Scale bar: A, C=275 µm. E=300 µm.

Development • Supplementary information

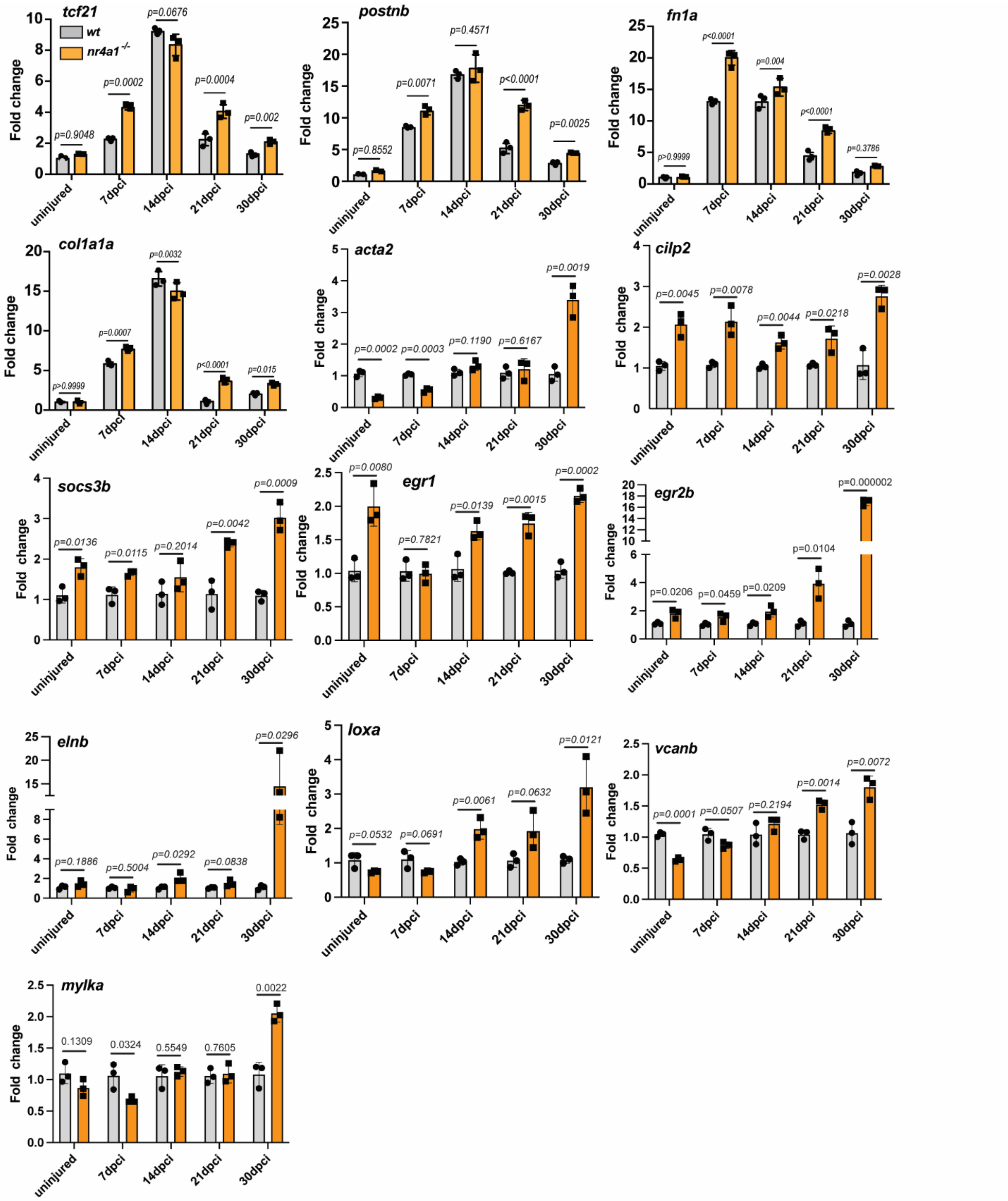

Fig. S10. Aberrant expression of ECM genes in *nr4a1* mutant upon cardiac injury

qPCR of both regenerative and profibrotic ECM genes in the hearts of indicated genotypes. Fold changes of *tcf21*, *postnb, fn1a* and *col1a1a* are calculated relative to the expression in uninjured wt hearts. Fold changes for the rest of genes are calculated relative to expression of control groups at different injured stages. Two-way ANOVA with Sidak test for multiple comparison correction. P-values<0.05 were considered to be statistically significant.

Development • Supplementary information

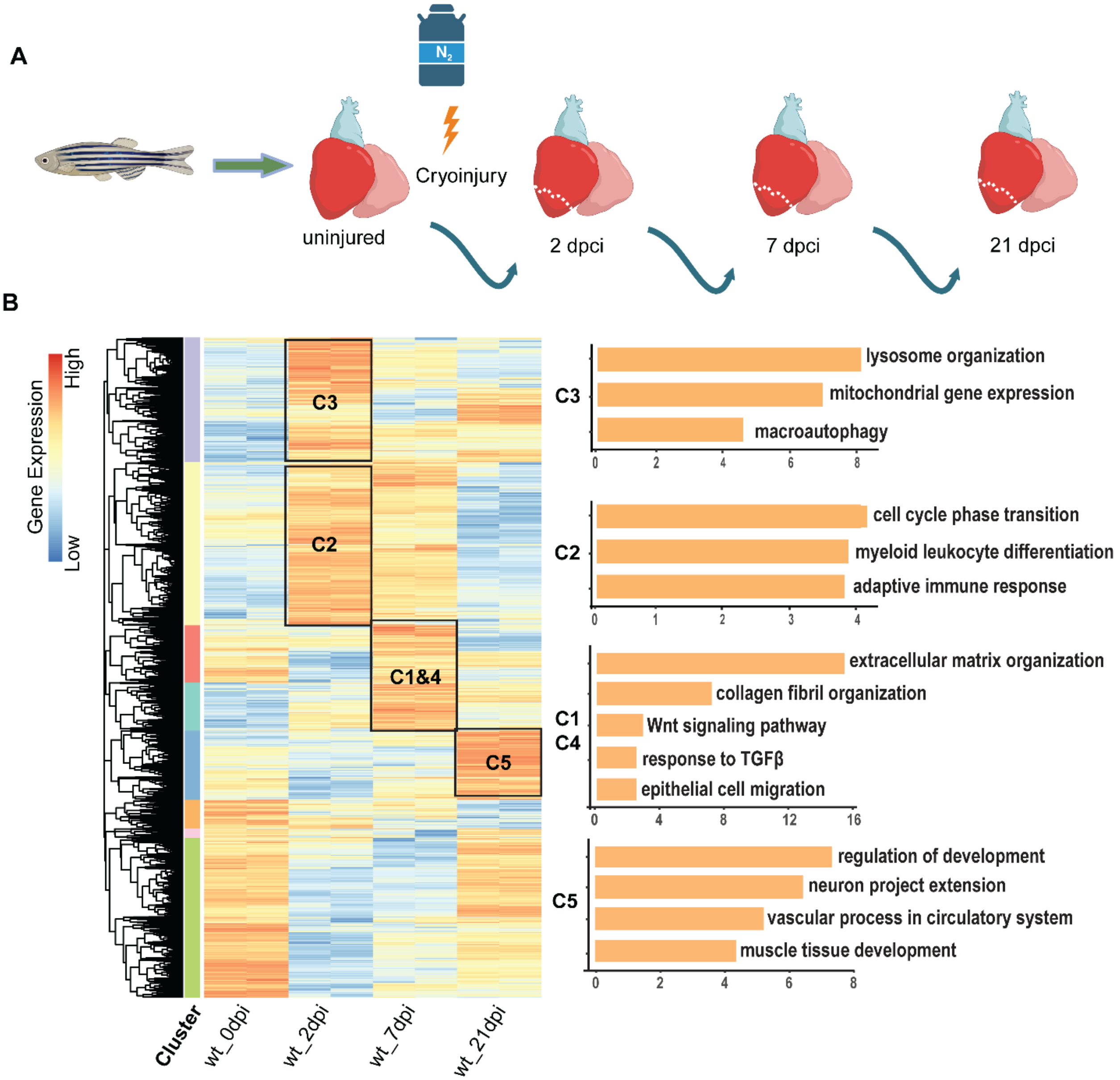

Fig. S11. Transcriptomic profiling of control and mutant hearts across multiple time points

(A) Schematic images showing the workflow for bulk RNA sequencing. Created in BioRender by Feng, D., 2025. https://BioRender.com/zwisodr. This figure was sublicensed under CC-BY 4.0 terms. (B) Hierarchical clustering heatmap of DEGs in control hearts across multiple time points with predicted GO terms listed in the right panels.

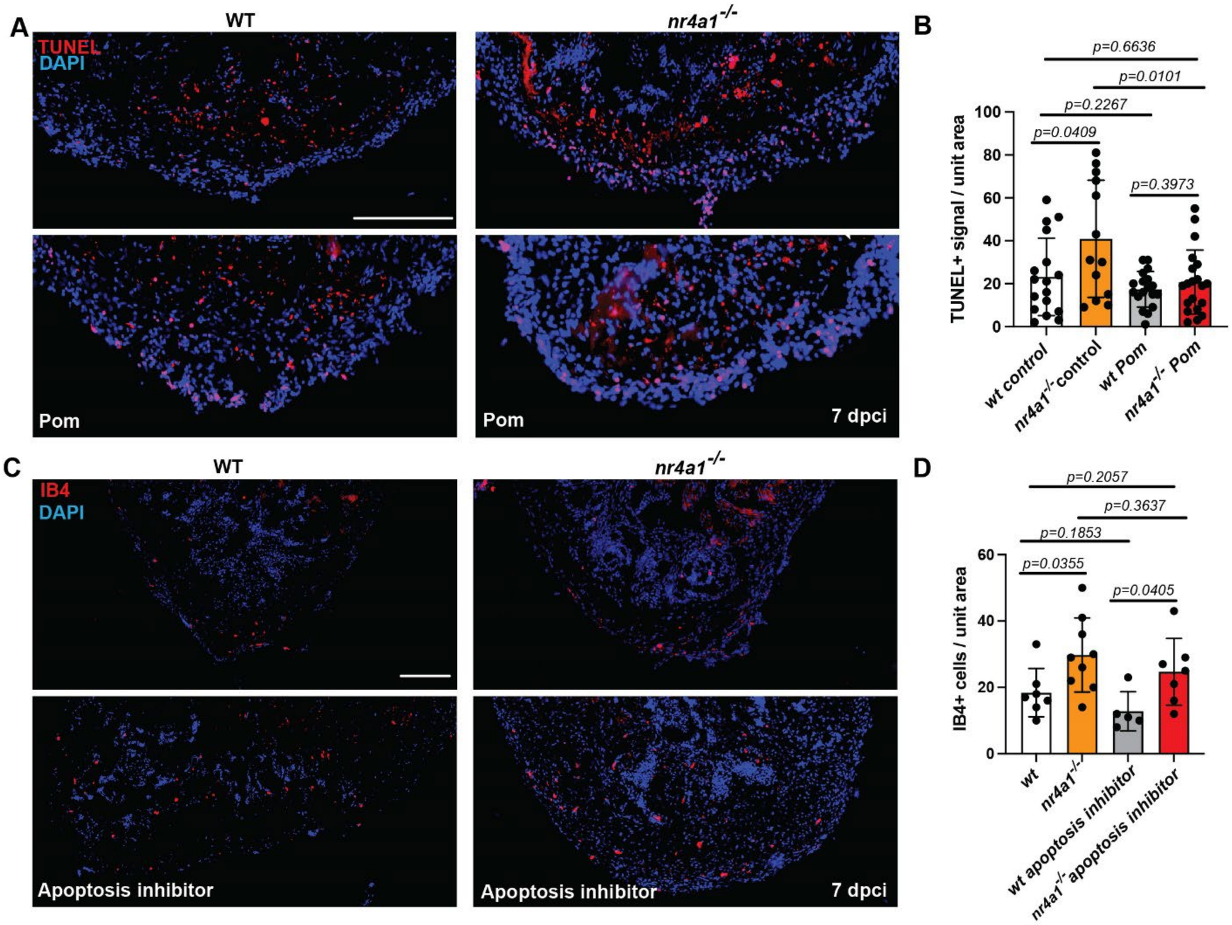

Fig. S12. Anti-inflammation treatment inhibits apoptosis in *nr4a1* mutant hearts

(A) TUNEL staining in wt and *nr4a1* mutants with Pom treatment at 7 dpci. (B) Quantification of TUNEL+ cells after Pom treatment at 7 dpci. (C) IB4 staining in wt and *nr4a1* mutants after apoptosis inhibitor treatment at 7 dpci. (D) Quantification of IB4+ cells after apoptosis inhibitor treatment at 7 dpci. The entire image in A and C represents a unit area. Symbols show the sample number. A two-tailed unpaired t-test is used. P-values<0.05 were considered statistically significant. Scale bar in A=275 µm, C=100 µm.

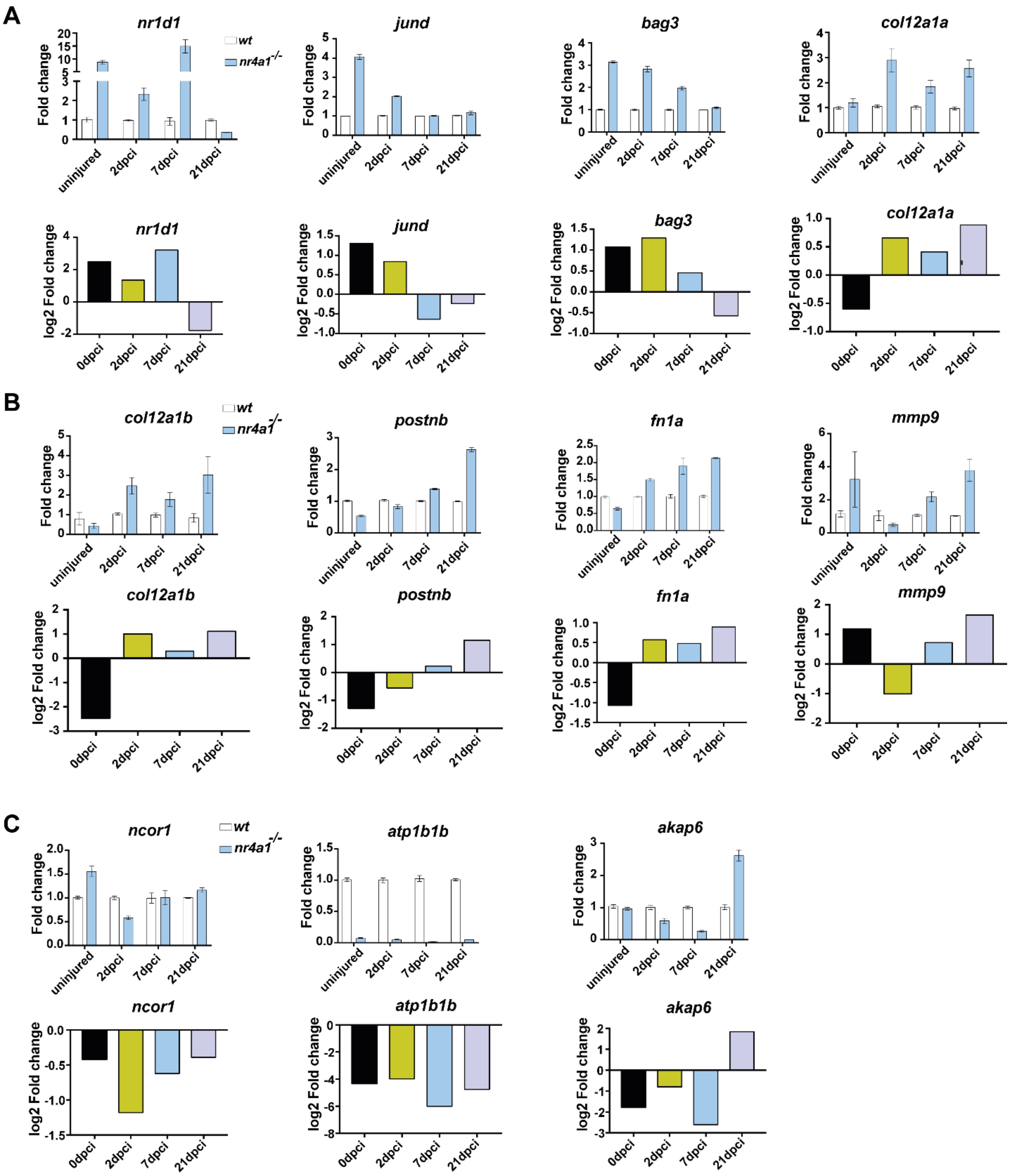

Fig. S13. Validation of the expression of the DEGs identified in transcriptomic profiling using qRT-PCR

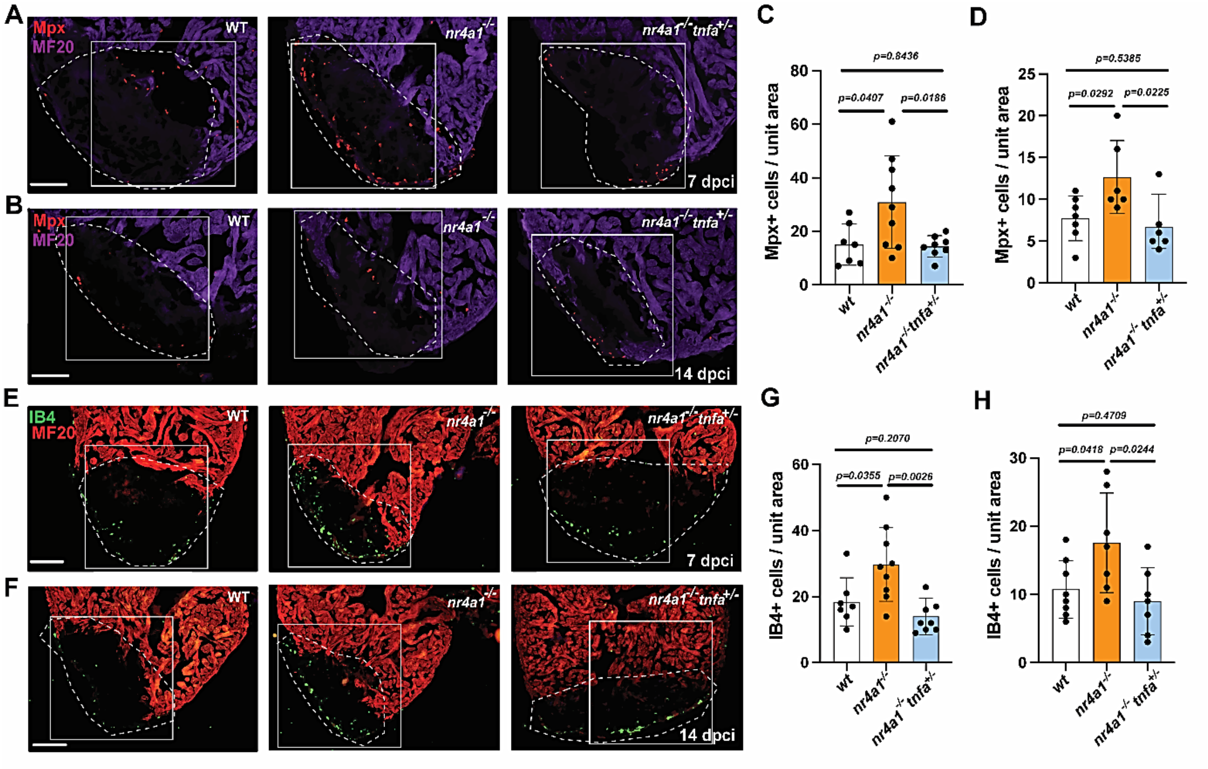

Fig. S14. ***tnfa***$^{+/-}$ **rescues the neutrophil and macrophage responses in** ***nr4a1***$^{-/-}$ **regenerating hearts**

(A, B) Representative cardiac section images of neutrophils distribution in three groups at 7 dpci and 14 dpci, respectively, stained with antibodies against Mpx; (C, D) Quantification of neutrophil numbers at 7 and 14 dpci, respectively; (E, F) isolectin-B4 (IB4) stained macrophages distribution at 7 dpci and 14 dpci, respectively; (G, H) Quantification of macrophage numbers at 7 and 14 dpci. Dash lines mark the injured area. Box regions show the approximate positions for quantification. A two-tailed unpaired t-test is used. P-values<0.05 were considered to be statistically significant. Scale bar =100 µm.

Table S1. The sequences of primers used in the study.

Available for download at
https://journals.biologists.com/dev/article-lookup/doi/10.1242/dev.204395#supplementary-data

Supplementary Materials and Methods

Quantification of staining and cell populations

All image analyses were performed using Fiji software. The percentage of the wounded area in AFOG staining and in MF20 staining at 30 dpci was quantified by outlining the region of interest in Fiji. For the regeneration score (%) in MF20 staining at 60 dpci, hearts were classified as fully regenerated (intact myocardial wall in the wounded area), partially regenerated (partially restored myocardial wall) or non-regenerated (minimal or no myocardial structure in the wounded area).

Cell numbers, including Nr4a1^{+} cells in TgKI(nr4a1-eGFP), mpx^{+} cells, IB4^{+} cells and GFP^{+} cells in TgBAC(tnfa:EGFP), Tg(lyz:EGFP) and Tg(tcf21:nucGFP) sections, were manually counted using Fiji. GFP^{+} cells in whole-mount Tg(lyz:EGFP) samples were quantified in the same way per unit area. Cardiomyocyte proliferation (PCNA staining) was quantified as the number of nuclei co-labeled with Nkx2.5 (red) and PCNA (green), appearing yellow, divided by the total number of Nkx2.5^{+} nuclei within defined unit areas shown in the figures.

For neutrophil quantification, the percentage in the wounded area was calculated as (number of neutrophils in the wounded area / total neutrophils per unit area) × 100.

When dense cell accumulation [e.g., in TgBAC(tnfa:EGFP) and Tg(mpeg1:EGFP)] made individual cell counting impractical, a threshold-based approach in Fiji was used. TIFF images were converted to 8-bit grayscale, the wounded area was outlined and duplicated, and threshold values were applied [90 for whole-heart TgBAC(tnfa:EGFP) and Tg(mpeg1:EGFP) samples; 40 for Tg(mpeg1:EGFP) sections]. Particles outside the selected area were cleared, and the ‘Analyze Particles’ tool was used to measure pixel areas in the wounded region and in the entire unit area. The percentage was then calculated as (pixels in wounded area / pixels in unit area) × 100.

Collagen deposition, *in situ* hybridization signals and α-SMA signals were quantified similarly using thresholding, with threshold values set at 20 for collagen, 88 for *in situ* hybridization, and 10 (30 dpci) or 15 (60 dpci) for α-SMA.

For TUNEL staining, red^{+} signals were manually counted in defined unit areas using Fiji.
